# The relevance of U.S. Strategic Highway Safety Plans in a future context

**DOI:** 10.1371/journal.pone.0223646

**Published:** 2019-10-07

**Authors:** Brett P. Hughes, Torbjorn Falkmer, Anna Anund, Melissa H. Black

**Affiliations:** 1 School of Occupational Therapy, Social Work and Speech Pathology, Curtin University, Perth, Australia; 2 Pain and Rehabilitation Centre, Department of Medical and Health Sciences, Linkoping University, Linkoping, Sweden; 3 Swedish Road and Transport Research Institute, VTI, Linkoping, Sweden; Tongii University, CHINA

## Abstract

While road safety in the United States (U.S.) has been continually improving since the 1970's, there are indications that these improvements are becoming increasingly difficult to sustain. Strategic Highway Safety Plans (SHSPs) are prepared by States to guide road safety management, however assessing the appropriateness of these plans remains a significant challenge, especially for the future in which they are to be applied. This study developed a new methodology to assess SHSPs from the perspectives of comprehensive system-based safety management and relevant future issues that can be applied before SHSPs are implemented, thereby avoiding long periods after implementation before assessing the appropriateness of the plans. A rating scale was developed and applied to assess 48 U.S. SHSPs against two key criteria: 1. a comprehensive framework for road safety, and 2. the anticipated changing, difficult and unpredictable nature of future transport and its context. The analysis concluded that current SHSPs have good national oversight with several strengths but were weak in some areas of content and did not address future challenges. Improvements are suggested to strengthen the plans’ thoroughness by being consistent with systems theory and practice, as well as ways that these SHSPs can be more resilient to future circumstances. Implementing the recommendations in this paper provides the opportunity to adopt a system-based safety management practice that has been successful in other hazardous industries. Doing so is expected to most efficiently and effectively continue the recent improvements to road safety, which is likely to be increasingly difficult otherwise.

## Introduction

**Fig 1:**
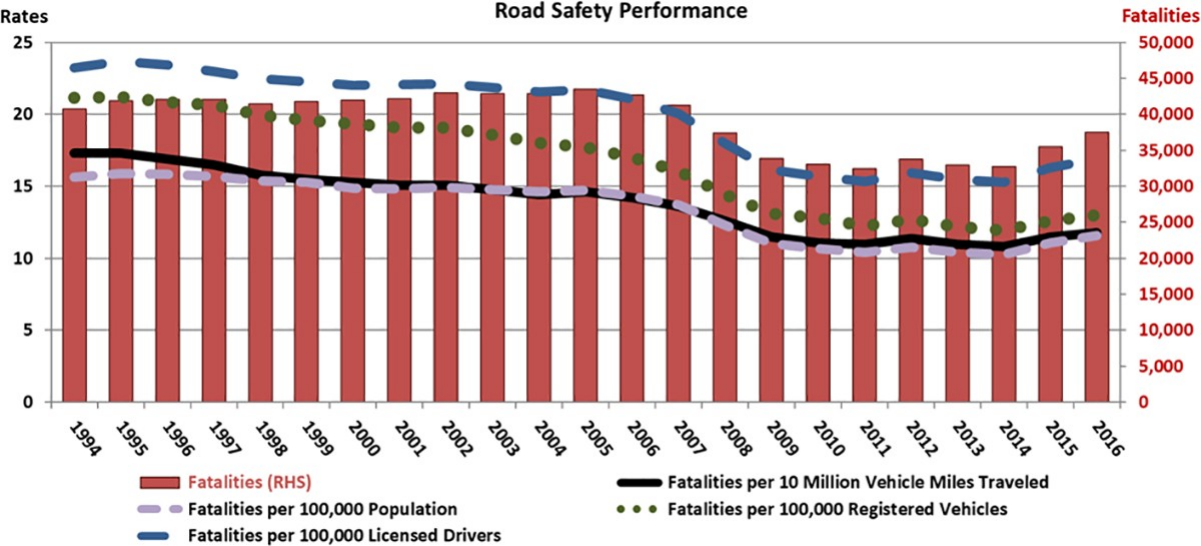
U.S. road fatalities and rates.

In the ten years ending 2016, 320,874 people died in traffic crashes in the United States (U.S.), and approximately 100 times as many were injured [1]. Fatality and injury rates resulting from crashes remained reasonably unchanged until 2005 before declining until 2010. Numbers then remained relatively constant until 2014 and have since risen up until 2016 [1], as shown in Fig 1. The total numbers and the rates against population, number of drivers, vehicles and miles travelled followed similar trajectories. The cause for concern is that road safety appears not to have improved in the five years up to 2016 and may even be deteriorating. Improvements in road safety have similarly discontinued in other developed countries [2,3], but it is difficult to determine the reasons underlying these patterns and how road safety can continue to be improved into the future.

To qualify for Federal road funds, U.S. States are required to develop, implement, evaluate and update a Strategic Highway Safety Plan (SHSP) that identifies and analyzes road safety problems and opportunities on all public roads [4]. However, assessing the appropriateness of SHSPs in efficiently and effectively achieving their objectives, especially for the future in which they are to be applied, is a key challenge. This paper describes the development and application of a new methodology to assess current SHSPs against two criteria that can be used prior to the SHSPs being implemented. The first set of criteria are the seven elements of a newly developed comprehensive framework for road safety management, based on systems theory and practice [5,6]. The second criteria represent the changes that are expected in the transport system and its context that are likely to affect road safety [4,7,8], including the changing and variable nature of future transport [6,9–11].

### Assessing road safety plans

Assessing the effectiveness and efficiency of road safety plans is problematic. The causal effect of a safety plan in achieving its objectives is difficult to demonstrate, partly due to the confounding or contributing factors, such as changes to the economic context [12–14]. Federal legislation requires SHSPs to be evaluated, because “*if an SHSP goal or objective is not met*, *the results may suggest a strategy is ineffective*, *or in some cases*, *the process for implementation did not go as planned and needs to be reconsidered*.” [15]. Evaluation makes judgments about the activities, characteristics, and outcomes of a program, to improve its effectiveness, and/or inform decisions about future programming, and has three levels of objectives: Process, Output and Outcome [16]. The evolution to current road safety management appears to have been successful in parts of Europe, but the evidence is difficult to interpret [17], because “*it is tempting to attribute changes in the time series of road safety measures to contemporary initiatives and intervention*. *This easy confidence in our ability to link effect to cause may be unrealistic and misdirect our actions*.” ([18], p45).

Several limitations exist in the current evaluation frameworks applied to assess the appropriateness, effectiveness, and efficiency of current road safety plans. Quantitative rating to assess safety policy and leadership has found considerable variation between U.S. States in countermeasure adoption and leadership, with each State having different strengths [19]. Furthermore, meaningful causal relationships between road safety management and outcomes were found to be difficult to identify, so it was concluded that there was no reliable relationship between road safety management tools and improved road safety outcomes, despite the obvious rationality and attractiveness of the proposition [20]. Perhaps critically, current SHSPs are evaluated sometime after implementation which may result in considerable funds being allocated to ineffective and inefficient plans. Significant time is required to collect and analyze data which may confound the validity of the assessment and also delays its utility, perhaps by so long that it is not very helpful [17,18]. Given the significant limitations associated with the current assessment of SHSPs, there is a strong case for constructive, critical assessments of the quality and value of SHSPs, as well as recommendations to make improvements to them.

### Road safety management development

Early approaches to road safety management focused on the key component parts of drivers, vehicles and roads (1920’s), for which engineering, enforcement and education (1930’s) was developed and applied to reduce crashes [6]. Road safety management subsequently evolved through several paradigms, described for example as i) Control of motorized carriage, ii) Mastering traffic situations, iii) Managing the traffic system, and iv) Managing the transport system [21]. These developments often reflected safety management from other domains, such as the epidemiological ‘host-agent-environment’ [22,23], the ‘human-machine-environment’ or sequence of causal events models [24–26].

Road safety management has evolved by modifying traditional approaches incrementally. U.S. road safety management continues to apply the 4E policy tools of Engineering, Enforcement, Education and Emergency response to road users, vehicles and roads [15], as is used in most developed countries, at least until a few years ago. In Europe and Australasia, road safety management has evolved most recently into Vision Zero or Safe Systems descriptions [27], which have incorporated an underlying moral argument and added the dimension of principles by which countermeasures are developed and applied. Despite these underlying differences, road safety practice at the operational level is the same in all cases, relying primarily on education, enforcement and engineering applied to drivers, vehicles and roads [12].

U.S. road safety management is strongly influenced by Federal legislation and direction, together with other nationally coordinated information [28,29]. The Federal Highway Administration (FHWA) recognizes many diverse issues that guide SHSPs, such as leadership, safety management, program delivery, consultation, coordination, system performance, corporate capacity and evaluation. Other countries have started to apply systems concepts to road safety, in the U.S. commonly known as Toward Zero Deaths (TZD) [3,19,29,30], but these concepts have not yet been thoroughly, widely or consistently applied in the U.S.

### Safety management in hazardous industries

Similar to road safety, safety management in hazardous industries evolved through several paradigms and approaches. However, in contrast to road safety, safety management in hazardous industries over the past 20 years has moved away from the human-machine-environment interface model and causal sequence analysis that considered parts in isolation [6,31] and has adopted and applied systems theory [32–36] developed from early applications in industry [5,24,37–40]. A sociotechnical system may be defined as “*an interacting combination*, *at any level of complexity*, *of people*, *materials*, *tools*, *machines*, *software*, *facilities*, *and procedures designed to work together for some common purpose*” ([5], p22). Systems theory was developed to overcome a mechanistic or ‘machine theory’ approach that “*attempted to analyse the vital process into particular occurrences proceeding in single parts or mechanisms independently of one another*.”, because “*the essence of the organism* (is seen) *in the harmony and co-ordination of the processes among one another*” ([34], p177).

Systems-based safety management emerged to counter the limitations of traditional techniques that did not provide sufficient levels of safety [31,38,39], as evidenced by several high profile catastrophes in diverse hazardous industries in the 1980’s. Industrial safety management, particularly in safety-critical industries, such as nuclear, aerospace and offshore petroleum industries found that linear, simplistic and reductionist models were inadequate to deal with complexity and uncertainty [11,31]. Linear models did not recognize all interactions, feedback or the dynamic nature of systems that do not always operate in equilibrium over time or even for short periods. This oversimplification ignored some of the numerous contributing component parts, participants, factors or other influences and neglected broader contributions from standards, regulatory regimes, business practices, economic factors or organizations. Reductionist approaches were found to be inadequate because they focused too narrowly on only a few parts of the system that affected the results, increasingly relying on detailed analyses of individual parts or actions in isolation. Ultimately, these approaches did not provide sufficient levels of safety.

Consequently, hazardous industries have progressively and thoroughly adopted system approaches to manage safety efficiently and effectively. Modern reliability engineering and safety management in complex safety-critical industries have developed and adopted sophisticated, multidimensional and comprehensive approaches based on systems theory [11,31,41–43]. As a result, current safety management in these industries is indeed based on systems theory and best practice that broadens the choice of policy tools that can be applied to all components (or parts) that comprise the system being managed [11,31,44,45]. In contrast to road safety, this approach also specifically recognizes the full range of participants (or actors), the partnerships (or relationships and interactions) within the system, and the necessary processes to efficiently and effectively achieve the purpose. These approaches also recognize the importance of the way that systems and organizations operate (or safety culture and climate) in affecting and improving safety outcomes [46–51].

The dynamic nature of systems has been recognized for some time [11,33], but the changing current and future contexts require new paradigms for policymaking incorporating a systems approach, because “*theories*, *models*, *philosophies*, *and methods stemming from an earlier era of scientific thought and developed for simpler*, *mostly physical systems are largely inapplicable*” for current circumstances ([33], p7).

The most dramatic and structural changes that occur to systems are described as ‘disruptions’ affecting society, the economy, industry or business, which are expected to escalate and occur faster in future [7,8,10,31,52–54]. New business models and applications are developing as different sectors interconnect and converge. Some specific changes have already altered transport and further, more substantial changes are expected to transform transport even more, such as automation. As transport changes, so does road safety, which needs to respond to accordingly, either to maximize the opportunities presented by these changes or to mitigate negative impacts. The context of road safety and its greater context has become increasingly volatile, uncertain, complex, and ambiguous (or VUCA) [9,55].

### A comprehensive systems-based framework for road safety

**Fig 2:**
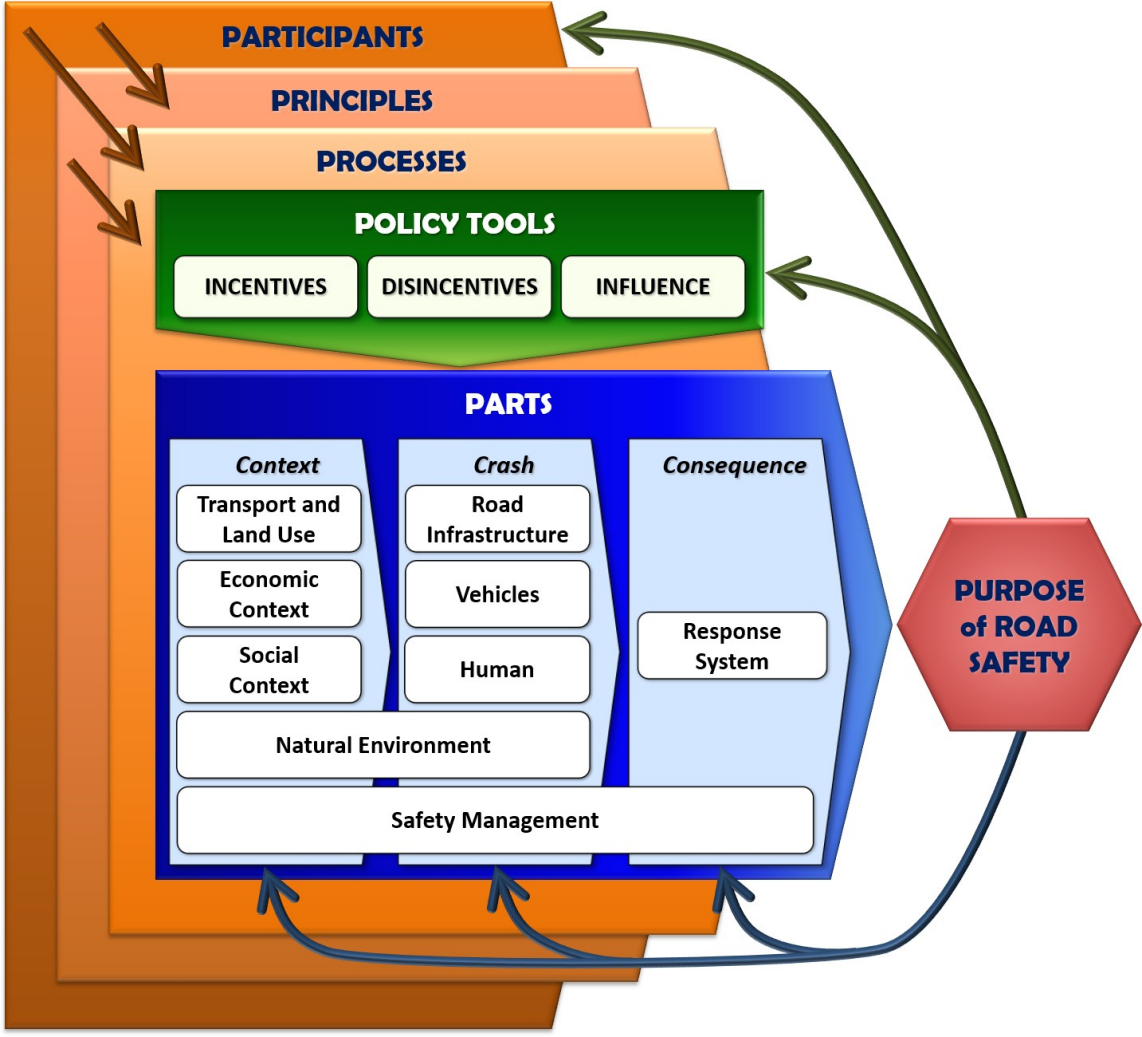
The 7P System framework for road safety management.

The potential for systems theory and practice to be applied to road safety has been recognized for many years but has failed to be thoroughly adopted and implemented [39,56–58]. In response, research to apply modern industrial safety management based on systems theory and practice to strategic road safety management has developed the comprehensive 7P System framework (Fig 2) [6] that can be summarized as: *Participants* use *processes* based on *principles* to apply *policy tools* to affect contributing component *parts*, in order to achieve a *purpose* (improved road safety). These all occur in complex interdependent *partnerships* or interactions within the system. The framework has similarities with other conceptual frameworks but is unusual in its breadth of content compared to previous conceptual frameworks for road safety [6]. The framework resulted from an extensive literature review of models relevant to road safety, so it incorporates many features of previous safety models [27]. In fact, the 7P System framework incorporates all dimensions of the most significant safety management approaches previously applied, viz. i) traditional road safety management, ii) Haddon’s original ten countermeasures [59], iii) all versions of the Haddon Matrix [60], iv) current U.S. national safety management, v) Vision Zero or Safe Systems and vi) contemporary systems-based safety management.

Consequently, this study developed a new methodology to assess SHSPs from the perspectives of comprehensive system-based safety management and relevant future issues while also overcoming the need to wait for long periods after implementation before assessing the appropriateness of the plans.

The 7P System framework incorporates many more policy tools, parts and participants than the content in current U.S. road safety management guidance or traditional road safety management [6,12]. Traditional road safety management has applied the 4E’s (Engineering, Enforcement, Education and Emergency response) to three parts (drivers, vehicles and roads), whereas the 7P System framework extends to ten policy tools applied to nine parts [6,12]. Table 1 summaries the differences between the traditional approach to road safety management that is practiced in the U.S. with the Policy Tools, Parts (or system components) and Participants (or actors) based on systems theory and approaches from other safety domains, as it would be applied to road safety management [6]. It also recognizes the wide range of Participants (or actors) that can affect road safety outcomes that are often neglected in managing road safety [58].

**Table 1:** Policy tools, parts and participants in traditional road safety management compared with the P7 System framework.

	Traditional U.S. Road Safety Management	7P System Framework
**Policy tools**	1. Education 2. Enforcement 3. Engineering. 4. Emergency response	**Incentives** 1. Funding & investment 2. Financial incentives, pricing & subsidies **Disincentives** 3. Regulation, enforcement, penalties & sanctions 4. Taxes, fees, levies & charges **Influence** 5. Leadership, integration, implementation & participation 6. Standards & guidelines 7. Behavior change 8. Skills, expertise, capability & professional practice 9. Industry change, competition & consumer choice 10. Innovation & research
**Parts** (system components)	1. Drivers 2. Vehicles 3. Road infrastructure	1. Safety Management **Context Phase** (pre-crash) 2. Transport and land use 3. Economic context 4. Social context 5. Natural environment **Crash Phase** 6. Road infrastructure 7. Vehicles 8. Humans **Consequence phase** (post-crash) 9. Response system (first responders, emergency services, emergency treatment, medication treatment, rehabilitation, adaptation, return to driving, monitoring)
**Participants**	*For consultation*, *representatives of*: 1. Governor 2. transportation and metropolitan planning 3. major modes of transportation 4. traffic enforcement 5. highway-rail grade crossings 6. motor carrier safety program 7. motor vehicle administration 8. nonmotorized users 9. other major Federal, State, tribal, and local safety stakeholders.	*Potential participants in all phases of activity* 1. Legislators 2. Government agencies (including all levels of government, regulators, courts, educators, researchers, police, road authorities, licensing authorities, safety agencies, emergency responders and health departments) 3. Industry and other groups 4. Operations and delivery companies and agencies (including transport users, transport companies and general industry) 5. Individuals and users (including families, employers, health care workers, transport users, and departments)

The 7P System framework incorporates all essential system dimensions including the full range of participants and processes (e.g. risk analysis and implementation) which are explicitly identified. The 7P System framework includes both feedforward and feedback partnerships (interactions or interrelationships) that are particularly numerous and complicated for road safety [61] but are ignored in road safety management. While Vision Zero includes valuable principles, they are not universally accepted or consistent. Vision Zero principles are different to those in other road safety approaches, such as the Dutch Sustainable Safety [62], so the 7P System framework allows for other principles that could be adopted as a basis, such as value for money or community acceptance.

The 7P System framework is consistent with frameworks in more general fields, such as management or community development, which it is intended to augment, not replicate. The framework is also consistent with other common management practices, [63] that cover financial management, strategic planning, operations management, legal issues, project management, communications and human resources. Other general frameworks, such as for community development [64], have clear goals and principles to guide practice, which could be useful if applied to road safety management because they are different to those provided by FHWA, American Association of State Highway and Transportation Officials (AASHTO) or TZD. U.S. Agency International Development (USAID) [64] also has very clear processes that are not included in traditional road safety management, and are recognized as essential in the 7P System framework. The USAID five R’s scope; Results, Roles, Relationships, Rules and Resources is also more extensive and detailed than traditional road safety management [65]. However, these approaches do not clearly describe the full range of policy tools and system parts that are relevant to any specific context or issue. By contrast, the 7P framework includes numerous specific systems components and policy tools that are not described or organized clearly elsewhere. While other frameworks and guidance material provide the opportunity for improving road safety management, the 7P System framework provides a broader overview as well as some detailed content that is not found in other frameworks. The 7P framework considers road safety from a less common, holistic and comprehensive approach which complements micro-level approaches and analyses that are more common, such as those used by Chen et al. [66] and Chen et al. [67]. Salmon and Lenné [58] adapted Rasmussen’s accident causation approach to describe seven predictions specifically relevant to road safety that are consistent with the 7P System framework but are more prominently and clearly stated. These relate to the contributions of many participants and contributing factors rather than a few, and the significance of relationships including vertical communication and feedback. They argue that taking account of these in road safety management requires a paradigm shift, that the 7P System framework is consistent with.

So, while the 7P framework has many similarities with other management and frameworks, some of which could be complementary, it has distinct advantages for this circumstance; i) it is specific to road safety; ii) it is thoroughly based in systems theory and practice, and iii) it incorporates more dimensions and concepts than other frameworks, including those from several other frameworks.

## Analytical approach

To counter the existing weaknesses of analyzing the appropriateness of SHSPs for a future context, this study describes a new method for examining and scoring SHSPs. This *ex ante* analysis overcomes the delay in assessing the appropriateness of SHSP’s which occurs with *ex post* analyses, examines SHSPs from a comprehensive systems perspective, and considers future contextual issues that will be relevant to the success of SHSPs.

The analytical basis for this study was based on the systems theory paradigm introduced above and the need to respond to future changes that have already been occurring but have accelerated more recently. Therefore, the present paper developed and applied a rating scale to assess 48 U.S. SHSPs against two key criteria: 1. a comprehensive framework for road safety, and 2. the anticipated changing, difficult and unpredictable nature of future transport and its context.

### 7P System criteria

Rating scales based on the 7P System framework were developed to assess SHSPs against a comprehensive systems approach to manage and improve road safety [6], as described in Table 2.

**Table 2:** Summary of 7P system criteria and scoring.

7P System criteria	Description	Concepts and indicative criteria terms
**1. Purpose** (outcomes)	Consequences of a system when it is functioning, or something of value that is produced or occurs as a result.	*Score 2*: goal, objective, target, aim, outcome targets, vision, mission. *Higher score*: targets for individual emphasis areas, TZD, extended analysis.
**2. Policy Tools**	Any specific intervention or countermeasure applied to improve safety including policies, programs and/or projects (e.g. pricing, education or regulation).	*Score 2*: engineering, enforcement, education, emergency response. *Higher score*: funding, investment, incentives, pricing, subsidies, fees, charges, leadership, integration, implementation, participation, behavior change, skills, expertise, capability, industry change, competition, consumer choice, innovation, research.
**3. Parts** (system components)	Subordinate components of a system (e.g. drivers, vehicles and roads in the road safety system).	*Score 2*: road users, vehicles, roads. *Higher score*: transport, land use, economy, society, natural environment, other users, crash response, safety management.
**4. Participants** (actors)	Any individual or entity that has the capability to affect road safety, including government, agency, association, company or individual person. Sometimes categorized as users or stakeholders.	*Score 2*: police, road authority, licensing authority, road safety agency. *Higher score*: additional participants (e.g. courts, educators, researchers, industry advocates & associations, community groups, general public, transport & other government agencies, companies, media, transport users, unions, crash responders, etc.).
**5. Principles**	A general rule to be followed, or moral value to be used as a guide or put into practice.	*Score 2*: AASHTO, safety management, TZD or Safe Systems principles *Higher score*: additional principles (e.g. innovation, administrative efficiency & effectiveness, resilience to future change, national consistency, practicability, operational & commercial efficiency & effectiveness).
**6. Processes**	Complementary activities thoroughly applied to achieve an outcome.	*Score 2*: FHWA or AASHTO processes, implementation, evaluation, data analysis, safety management, research, strategic planning, project design/implement/operate, communications, evaluation. *Higher score*: other processes (e.g. in-depth crash investigation, safety/risk management, scenario assessments, benefit-cost assessment, evaluation, etc.)
**7. Partnerships** (relationships)	The interactions between actors, policy tools, components and outcomes, which may be positive or negative, forwards or feedback.	*Score*: *2*: integrate, connect, interconnect, interact, synergy, complement, conflict, dependency, collaboration, etc. *Higher score*: broader range and description or greater level of detail.

### Future changes criteria

SHSP’s need to take account of the future context to ensure they are relevant during the period of their applicability, given the significance and the unpredictability of these changes. The FHWA notes four key changes influencing the future operating environment: Key Influencing Trends, Highway Industry Trends, Highway Transportation Trends and Strategic Issues [68]. While there are numerous commentaries about future changes, several key topics commonly arise, particularly automation and other innovative applications enabled by electronic, information and communications technologies (EICT) [7,8,10,31,52–54].

Automation in road transport has evolved through several phases including Intelligent Vehicle Highway Systems and transport telematics into what is commonly called Intelligent Transport Systems (ITS) [6,69]. Automation in vehicles is not new but the opportunities provided by EICT have resulted in numerous applications for engine and transmission management, comfort, driver information, driver assistance and control systems in modern vehicles. It is widely expected that automation will dramatically change road safety by eliminating many driver errors, but it will also change productivity, ownership, privacy, data, terrorism and other outcomes, as has occurred in other industries. Road transportation automation is currently focused on vehicle control systems that do not have the same applicability across all modes or users, particularly pedestrians and cyclists [70], so the benefits will not be evenly distributed or wide-ranging. Systems theory and practice also suggests that new technologies and applications will introduce new failures that will need to be managed, due to increasing complexity and the fact that technology application maturity takes time [31].

More widely, new business models are disrupting traditional commercial enterprises. The sharing economy (e.g. Airbnb, Uber) and other new information and transaction enterprises have emerged extremely quickly over the last few years [71]. These businesses are responsive to evolving consumer preferences, particularly demands for services that are easier, cheaper and faster. These include transport preferences of younger people, diverse urban lifestyles or different demands of aging populations compared to the past. In transport, new business models are converging with new technologies to service different transport user demands or preferences, one commonly described example of which is Mobility-as-a-Service (MaaS) [8,69,72].

Another aspect of future circumstances is the difficult and variable nature of conditions, which continues to be more changeable, unpredictable and difficult to manage [10,11,73]. The historical environment that has been simple, stable, clear and certain, as mentioned, can no longer be described as such [9,55]; "*Organisations today are under stress from a number of dynamic factors in their environment*, *such as technological changes*, *globalization*, *and market conditions*. *Modern socio-technical systems are characterized by increased complexity and coupling*, *and are as a consequence increasingly intractable*." ([55], p 955). These conditions make intended outcomes more difficult to achieve, requiring more integration and collaboration and thus a more robust and comprehensive framework and practice [31]. Participants, factors, relationships and feedback are not static for predictions and planning for road safety management, but change over time [58], which necessitates a paradigm shift in road safety management that takes these dynamic factors into account.

Modern safety management in safety-critical industries takes into account that the future will be different in nature compared to present circumstances. In road safety, various analyses attempt to determine the impact of actions in isolation, including before-and-after studies, cost-benefit analyses or other micro-level assessments. However, by assessing the impact of road safety plans *as a whole*, information will be generated that can be used for performance measurement and understanding the success of the plans (or lack thereof). The problem is that these assessments often assume steady-state conditions that are not reasonable in these changing circumstances. Consequently, relying on analysis that is based on historical information and perspectives introduces a risk that a plan will not suit the future conditions [18,20]. Instead, suitable processes need to ensure that plans are relevant to the future, such as alternative analytical techniques [74,75] including real options analysis [76], scenario analysis, systems dynamics and cognitive workplace analysis [31,43,76–78].

Future Changes criteria for the assessment in this study are based on the expected changes in the transport system and its context, that are likely to affect road safety [6,68] as described above and summarized in Table 3.

**Table 3:** Summary of future changes criteria and scoring.

Future changes criteria	Description	Concepts and indicative criteria terms (including new, different, future, change)
**1. New technologies**	New electronic, information, communications or other technology applications or vehicle types that change road transport.	*Score*: *2*: electronic, ITS, automated, driverless, autonomous, disrupt, big data, innovation, etc. *Higher score*: broader description or greater level of detail.
**2. New markets and business models**	New ways that businesses operate commercially, or new transport market delivery structures that change the way that road transport broadly operates.	*Score*: *2*: mobility-as-a-service, market, business, demand, mobility, etc. *Higher score*: broader description or greater level of detail.
**3. Different consumer demands**	Changing consumer preferences or demands, or new markets that change the demand for transport.	*Score*: *2*: consumer, preference, choice, demand, etc. *Higher score*: broader description or greater level of detail.
**4. Nature of the future**	Continuing movement away from the previous context that has been simple, stable, clear and certain.	*Score*: *2*: volatile, uncertain, complex, ambiguous, scenario, future, predict, change, difficult, etc. *Higher score*: broader description or greater level of detail.
**5. Future situation assessment**	Clear, accurate and considered appreciation of the future situation.	*Score*: *2*: trend, context, estimate, future, forecast, model, predict, economic/ social/ environmental context or effects, etc. *Higher score*: broader description or greater level of detail.

## Methods

Current SHSPs from the U.S. were downloaded from the States’ websites and assessed according to the seven 7P System criteria and the five Future Changes criteria, similar to previous comparative analysis based on a rating scale [19,79]. Most plans were five years old or less while three plans were older, and two States did not have SHSPs available. Most plans were valid for five years.

A five-point scale was developed and used to rate the extent to which the SHSPs reflect the assessment criteria that are summarized in Tables 2 and 3. Keywords and indicative search terms were selected according to the concepts relevant to each specific criterion, and the basic scoring scale is as follows:

0—keywords or concepts not mentioned (i.e. brief text only),
1—keywords or concepts directly *or* indirectly mentioned, *and* not directly discussed (i.e. longer text),
2—keywords or concepts mentioned and briefly discussed *or* addressed (i.e. including a response to the criteria),
3—keywords or concepts discussed *or* has actions to address,
4—keywords or concepts thoroughly discussed *and* have comprehensive actions to address.

A central mark of ‘2’ represents that the SHSP’s inclusion of the criteria was minimally adequate. To maximize the reliability of the scores, the scales were a) explicitly described with keywords and search terms, which minimizes inconsistencies between the rating of different SHSPs, and, b) rated in all cases by the first author who has forty years’ experience in road safety and transportation policy, planning and research. To avoid sampling issues, all 48 available SHSPs were assessed. Each of the SHSPs was individually read and notes taken regarding the content relevant to the criteria to score each criterion and maximize the consistency of analysis and description of qualitative information. Examples of content that resulted in a higher score were noted to illustrate superior quality plans and provide guidance for improvements. The scores were noted, as provided below, before being analyzed and interpreted as a whole group. The analysis involved basic descriptive statistical analysis including calculation of the mean and standard deviation of the ratings provided to each criterion. Qualitative descriptions were also extracted to enable interpretation, discussion, and conclusions. The data, analysis and results were provided to whole study team who are highly qualified and experienced in road safety research and analysis. All scores, the important qualitative descriptive information and the results are provided below.

## Results

Seven 7P System criteria were assessed, where a score of '2' represented a minimum acceptable pass consistent with the comprehensive approach based on systems theory and practice described above. This provided 336 individual scores, as summarized in Table 4. A score above a minimum acceptable level of two for these seven framework criteria as a whole was achieved by 21 SHSPs, with an average score of 2.05 for all SHSPs combined. There were only 28 individual maximum scores of four, 68 scores of three and 89 scores less than two. These numbers equate to 28% of scores above a minimum acceptable level, 45% at the minimum acceptable level and 27% below an acceptable level. The highest average scores were 2.46 for purpose and 2.35 for policy tools, while the lowest average scores were 1.13 for principles and 1.92 for processes. Only four SHSPs scored above an average of three for the 7P System criteria, while 27 SHSPs scored below an average of two, indicating they were basic and inadequately described a comprehensive framework.

**Table 4:** Summary of 7P system criteria assessment.

7P System criteria	Examples from the highest scored plans
**1. Purpose** Average Score: 2.46 Range: 0 to 4	Targets for the total numbers fatalities and serious injuries are required, plus these outcome measures versus rates (population, motor vehicle, VMT). Vision and Mission statements to guide direction and action (California). Targets for individual Emphasis Areas plus more specific targets including types of road users (Georgia & Arkansas). TZD as a basis and comparing goals to recent trends (South Carolina).
**2. Policy Tools** Average Score: 2.35 Range: 1 to 4	Leadership, factors for each individual emphasis area. Education of other participants including technology consumers, seek additional required funding, encourage transit use, safety management, trials of new anti-distraction technologies, medical professionals to inform drivers of the effects of medications (Texas). Change court processes and train judges, child restraint inspection stations, safety technicians and fitting stations for low-income families. Bucks for Buckles joint event to award financial incentives for properly restrained occupants. Crash reconstruction training and Operation Lifesaver for railway level crossings. (Kansas). Increase State excise tax on beer, require mandatory motorcycle insurance cover, provide quality data, analysis and tools to customers, provide incentives for older drivers who use alternative modes of transportation, Monitor ignition interlock manufacturers and installers to ensure the continued viability and validity of the program (Washington). Educate employers about adopting safe driving programs or policies, encourage insurance companies to expand safe driving incentive programs, engage diverse stakeholders, including dealerships and manufacturers, in promoting new technologies, promote technologies that improve community mobility, recognize that streets should safely accommodate all road users, develop and evaluate evidence-based fitness-to-drive assessment tools (Missouri).
**3. Parts** Average Score: 2.02 Range: 1 to 3	Each individual Emphasis Area has an analysis of contributing factors. High-risk road users, special vehicle types, behaviors and locations are identified (Ohio). Population, economy, transport activity, State & local issues, all modes, freight. Built environment, lighting, broader Transport Plan context, travel demand and consumer choices (Florida). Weather, non-motorized users, trucks, transit, incident management, railway crossings, transit, animals, traffic incidents, work zones (Arizona).
**4. Participants** Average Score: 2.29 Range: 1 to 4	Coalitions for Emphasis Areas: lane departure and intersections, impaired driving, pedestrian and bicycles, safe mobility for life, motorcycles, teen safe driving, traffic records coordinating committee, work zones (Florida). Dept. of Public Health, agencies responsible for transporting children, educational professional and child-care providers, anti-drug community support group, hospital staff discharging newborn children, parents, courts and legal system staff, local community task forces, schools, community groups, other transport and safety professionals, military base commanders, non-white populations at risk, Spanish speaking groups & other minorities (Georgia). Local and State law enforcement, and community groups, NHTSA, other Departments (Health, Environment, Revenue, Investigation, Attorney General, DUI Impact Center) (Kansas).
**5. Principles** Average Score: 1.13 Range: 0 to 4	Focus on priority areas, data-driven, goal-focused, implement proven strategies, change culture, integrate with other transport and safety plans at the state, regional, and local levels, ongoing monitoring and reassessment (Virginia). Facilitate partnerships, commit resources, innovate, break institutional barriers, communicate to partners, champion the cause, improve data, focus on high-risk behaviors, shared responsibilities (Ohio).
**6. Processes** Average Score: 1.92 Range: 0 to 4	Marketing, data management, performance monitoring and management, SHSP review and revision, research, planning and collaboration, inter-jurisdictional cooperation, implementation, management roles and responsibilities, administration (coordination, review, planning, facilitation, evaluation, communication, reporting) (Arizona). Sophisticated statistical crash analysis, organization formation, thorough implementation management, comprehensive evaluation (Ohio). Quality data analysis, linkages, tools, resourcing and communication. Collision, ticket and records systems. Evaluation, problem diagnosis, investment decisions (Washington).
**7. Partnerships** Average Score: 2.17 Range: 0 to 4	Integration with other multifaceted transport and State plans, each Focus Area includes actions and indicators complementary to other areas, interactions between participants actively investigated and collaborations described (Minnesota). Multi-agency initiatives and task forces, multidisciplinary and multifaceted programs with collaborations between diverse partners for Emphasis Areas, cross-training between multiple disciplines (Missouri). Co-benefits described for each action, stakeholders’ clear roles and responsibilities, links to other transport plans and levels of government, links with local government commissions, committees, and other affiliated groups (Oregon).

Five Future Changes criteria representing future conditions in which the SHSPs are expected to be applied were assessed, where a score of '2' represented a minimum acceptable score relevant to expected future conditions and suitable responses. This provided 240 individual scores, as summarized in Table 5. There were only six scores of four, only five scores of three and 11 scores of two, with the clear majority of scores (218) below a minimum acceptable score. These numbers equate to 4.6% of scores above a minimum acceptable level, 4.6% at the minimum acceptable level and 91% below an acceptable level. Only six of the SHSPs achieved a total average score above one, well below the acceptable level of two for these five criteria, with an average overall score of an extremely low 0.39 for all SHSPs. The highest score of 1.10 was for new technologies, while all other criteria scores averaged below 1.0. None of the SHSPs reflected the future situations to any degree of adequacy, with 33 SHSPs scoring zero in at least four Future Changes criteria.

**Table 5:** Summary of future changes criteria assessment.

Future Changes Criteria	Examples from the highest scored plans
**1. New technologies** Average Score: 1.10 Range: 0 to 4	Autonomous vehicle program with partnerships for research, plans, processes and public education. New technologies for ignition interlocks, impaired drivers, ITS, traffic signals, crash investigations (Virginia). ITS, new technologies for impaired driving warning, traffic signal approach warning and conspicuity, assistance for older drivers, driving performance feedback, truck drivers, dynamic speed and warning signs, pedestrian and rail crossings, electronic reporting and enforcement systems (Illinois). New technologies for EMS response, road condition, seat belt and speed warning, and other collision avoidance, level crossing enforcement, incident management, radio interoperability, data reporting and analysis (Missouri).
**2. New markets and business models** Average Score: 0.10 Range: 0 to 2	New mobility management (e.g. phone apps, car-sharing services, autonomous vehicles, etc.) providing new services for older adults, people with disabilities, and individuals with lower incomes (Missouri). Emerging private sector Mobility-as-a-Service (MaaS) options for seniors and other drivers (Pennsylvania). New transportation (e.g. car sharing and transportation network company services) with the widespread use of smart phones and other mobile devices (Oregon).
**3. Different consumer demands** Average Score: 0.06 Range: 0 to 1	Non-auto use (transit boardings, walking, cycling), changes in travel demand and transport choices. Changing commercial vehicle operation and demand (e.g. shopping) (Florida). Changing transport, lifestyle and residential preferences (Oregon).
**4. Nature of the future** Average Score: 0.08 Range: 0 to 1	Need to accurately identify and monitor changing environmental, behavioral, and vehicular factors, and more sophisticated statistical methods to monitor and predict outcomes. (Washington). More complex and integrated transportation systems need expanded and strong partnerships (Oregon). Complex tasks and issues, different future transport and context including consumer travel preferences, use of technology, travel choices etc. (Florida).
**5. Future situation assessment** Average Score: 0.63 Range: 0 to 4	Economic factors, capital and safety spending, vehicle fleet characteristics, and safety laws to predict risk with VMT to predict fatalities. Employment, fuel prices, vehicle crashworthiness and safety features to set targets. (Texas) Uncertainties role in fatalities, (economy, changes in legislation, funding priorities, and safety initiatives). Sophisticated mathematical model estimates confidence limits for future trends to inform target setting. Graphs, trend lines and confidence bands for performance indicators (Oklahoma). Future changes: aging and diverse population, more people in urban areas, transportation technology, health impacts, more global competition. 2020 crash projections for six key performance indicators (Minnesota).

**Fig 3:**
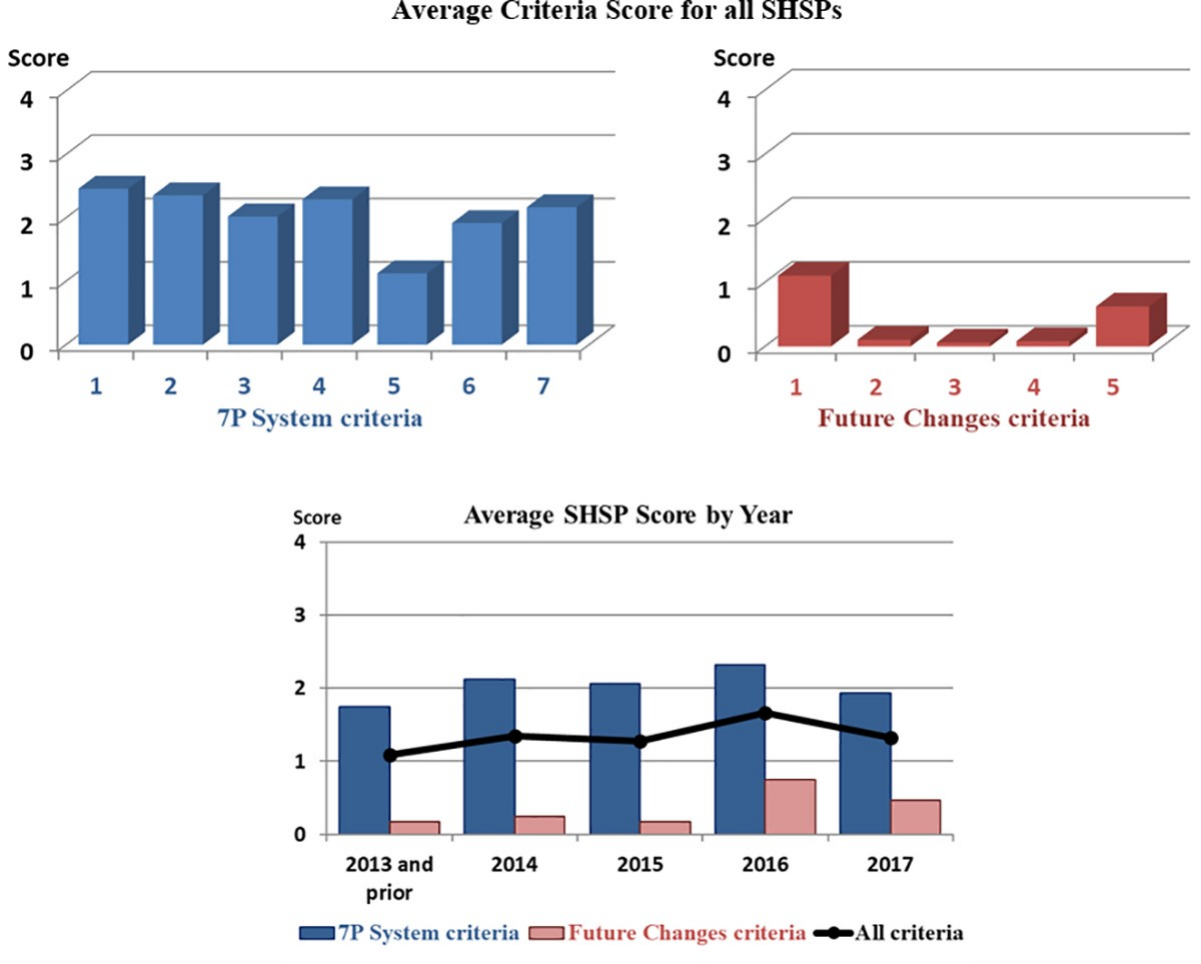
Average scores for SHSP assessments.

Fig 3 summarizes the assessment scores for the 7P System criteria and the Future Changes criteria separately, and all criteria together. This illustrates the moderate level of the scores against the 7P System criteria which ranged between 1.13 and 2.46, with an overall average of 2.05. The scores for the Future Changes criteria were much lower, ranging between 0.06 and 1.1, with an average score of 0.40. The scores varied slightly across the years ranging between 1.09 and 1.67. The SHSPs may be improving slightly over time, particularly for Future Changes criteria, but any change is likely to be marginal.

**Fig 4:**
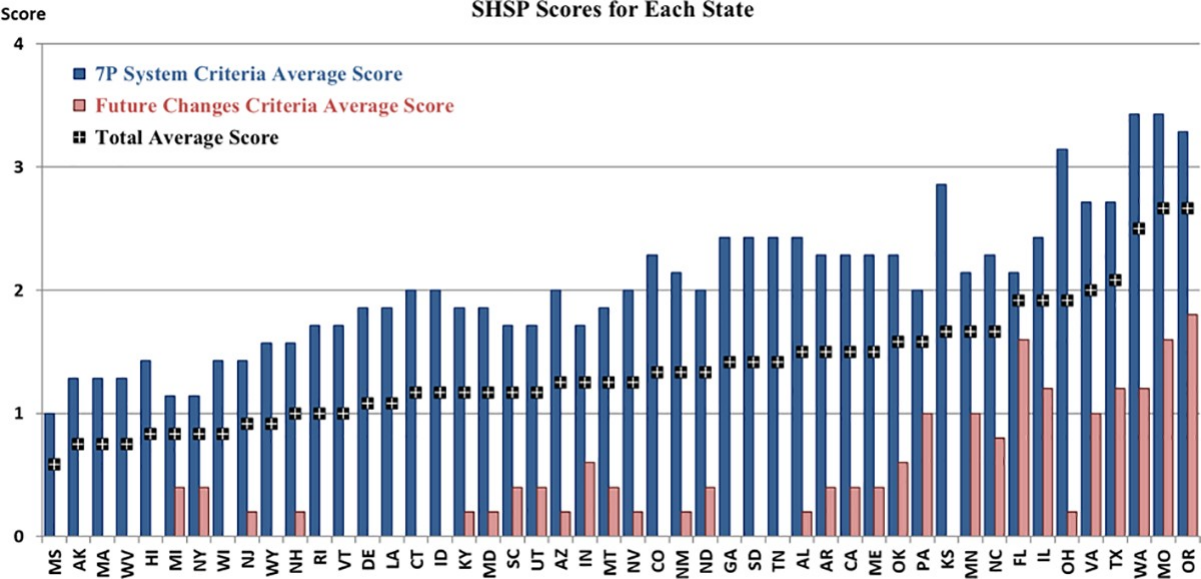
Individual SHSP assessment scores.

Table 6 summarizes the assessment for each individual SHSP which are illustrated in Fig 4. As a total, only four SHSPs achieved a minimum acceptable score of two as an average across all 12 criteria. Of the 576 individual scores overall, this equates to 19% of all individual scores above a minimum acceptable level (3 or 4), 28% at minimum acceptable level (2) and 53% below an acceptable level (0 or 1).

**Table 6:** Summary of individual SHSP assessment scores.

Year	SHSP No.	Overall Average	7P System Criteria								Future Changes Criteria					
			1.	2.	3.	4.	5.	6.	7.	Average	1.	2.	3.	4.	5.	Average
**2013 or before**	1	**1.00**	2	2	1	1	2	2	1	**1.57**	1	0	0	0	0	**0.20**
	2	**1.00**	3	2	2	2	0	2	1	**1.71**	0	0	0	0	0	**0.00**
	3	**1.50**	1	3	3	3	2	2	3	**2.43**	1	0	0	0	0	**0.20**
	4	**0.75**	0	2	2	2	1	1	1	**1.29**	0	0	0	0	0	**0.00**
	5	**0.75**	3	1	1	2	0	1	1	**1.29**	0	0	0	0	0	**0.00**
	6	**0.83**	1	3	2	2	0	1	1	**1.43**	0	0	0	0	0	**0.00**
	7	**1.33**	1	3	2	3	0	3	2	**2.00**	1	0	0	0	1	**0.40**
	8	**1.58**	3	2	2	2	2	2	3	**2.29**	0	0	0	0	3	**0.60**
**2014**	9	**0.58**	1	1	1	1	1	1	1	**1.00**	0	0	0	0	0	**0.00**
	10	**0.83**	3	2	2	2	0	1	0	**1.43**	0	0	0	0	0	**0.00**
	11	**1.25**	2	2	2	2	0	4	2	**2.00**	1	0	0	0	0	**0.20**
	12	**1.33**	2	3	2	3	2	2	2	**2.29**	0	0	0	0	0	**0.00**
	13	**1.42**	3	3	2	2	1	3	3	**2.43**	0	0	0	0	0	**0.00**
	14	**1.42**	3	3	2	2	1	3	3	**2.43**	0	0	0	0	0	**0.00**
	15	**1.67**	2	2	2	2	1	2	4	**2.14**	2	0	0	0	3	**1.00**
	16	**1.67**	2	2	2	3	1	3	3	**2.29**	2	0	0	0	2	**0.80**
	17	**1.92**	4	3	3	3	3	4	2	**3.14**	1	0	0	0	0	**0.20**
**2015**	18	**0.92**	1	3	1	1	2	1	1	**1.43**	1	0	0	0	0	**0.20**
	19	**1.08**	3	2	2	2	1	1	2	**1.86**	0	0	0	0	0	**0.00**
	20	**1.17**	2	3	2	2	1	2	1	**1.86**	1	0	0	0	0	**0.20**
	21	**1.17**	2	3	2	1	0	2	2	**1.71**	1	0	0	0	1	**0.40**
	22	**1.42**	4	2	2	4	0	2	3	**2.43**	0	0	0	0	0	**0.00**
	23	**1.50**	4	2	2	2	2	2	2	**2.29**	1	0	0	0	1	**0.40**
	24	**1.67**	4	4	2	4	0	2	4	**2.86**	0	0	0	0	0	**0.00**
**2016**	25	**1.17**	3	2	2	2	2	1	2	**2.00**	0	0	0	0	0	**0.00**
	26	**1.17**	3	2	2	2	0	1	3	**1.86**	1	0	0	0	0	**0.20**
	27	**1.17**	1	2	2	2	1	2	2	**1.71**	2	0	0	0	0	**0.40**
	28	**1.25**	1	2	2	3	0	2	2	**1.71**	3	0	0	0	0	**0.60**
	29	**1.25**	2	2	2	2	1	2	2	**1.86**	0	0	0	0	2	**0.40**
	30	**1.25**	3	2	2	3	0	2	2	**2.00**	0	0	0	0	1	**0.20**
	31	**1.33**	3	3	2	1	2	2	2	**2.14**	1	0	0	0	0	**0.20**
	32	**1.92**	1	2	3	4	0	1	4	**2.14**	4	1	1	1	1	**1.60**
	33	**2.50**	3	4	3	3	3	4	4	**3.43**	3	0	0	1	2	**1.20**
	34	**2.67**	3	4	3	4	4	2	4	**3.43**	4	2	0	1	1	**1.60**
	35	**2.67**	4	3	3	4	2	3	4	**3.29**	4	1	1	1	2	**1.80**
**2017**	36	**0.75**	2	2	2	1	0	1	1	**1.29**	0	0	0	0	0	**0.00**
	37	**0.83**	1	1	1	1	1	1	2	**1.14**	1	0	1	0	0	**0.40**
	38	**0.83**	2	1	2	1	0	1	1	**1.14**	2	0	0	0	0	**0.40**
	39	**0.92**	2	2	2	2	0	2	1	**1.57**	0	0	0	0	0	**0.00**
	40	**1.00**	3	2	2	2	1	0	2	**1.71**	0	0	0	0	0	**0.00**
	41	**1.08**	3	1	2	3	1	1	2	**1.86**	0	0	0	0	0	**0.00**
	42	**1.17**	3	2	2	2	2	2	1	**2.00**	0	0	0	0	0	**0.00**
	43	**1.50**	4	2	2	2	1	2	3	**2.29**	1	0	0	0	1	**0.40**
	44	**1.50**	2	2	2	3	2	2	3	**2.29**	1	0	0	0	1	**0.40**
	45	**1.58**	1	2	2	2	2	3	2	**2.00**	3	1	0	0	1	**1.00**
	46	**1.92**	4	3	2	3	1	1	3	**2.43**	4	0	0	0	2	**1.20**
	47	**2.00**	4	3	2	3	3	2	2	**2.71**	4	0	0	0	1	**1.00**
	48	**2.08**	4	4	2	2	2	3	2	**2.71**	2	0	0	0	4	**1.20**

## Discussion

This study developed and applied an assessment framework for SHSPs guided by a comprehensive systems-based framework for road safety and future issues. The application of this framework to 48 existing SHSPs demonstrates the utility of this framework in assessing the appropriateness and indicative effectiveness of future SHSPs using an *ex ante* approach.

There is a strong national multiagency approach to SHSPs with several sources providing support and guidance which includes Federal legislation, guidelines, oversight, monitoring and review, primarily by the FHWA. AASHTO and other organizations or collaborations, such as TZD, also provide additional support, although a greater vertical and horizontal integration at the individual State SHSP level would be beneficial [58]. Complying with this multiple agency guidance would result in SHSPs that would be at least adequate for most 7P System criteria, however the results of this study suggest that not all SHSPs appear to do so, indicating that many SHSPs are failing to adequately address guidance material. A number of potential factors may influence the application of guidance material in the development of SHSPs. For example, accessibility or knowledge of how to adequately utilize guidance and other supports may hinder its inclusion in SHSPs. In addition, our assessment describes other system criteria that are not well covered by Federal guidance, such as safety principles and processes. There are also other emerging and innovative concepts, such as Safety Culture [6,29,80–82], that are not included in guidance material. So, there is a risk that States could do as little as necessary to comply with Federal requirements to qualify for funds without necessarily producing the most efficient and effective SHSP. If so, strong Federal oversight could actually limit innovation. Continued effort is required to further improve SHSP guidance at the Federal or joint national level collaboratively and at the State level as SHSPs are developed and implemented. Investigating these potential factors was not within the scope of this study but warrants additional exploration in order to address these potential concerns.

Federal requirements include evaluation and implementation processes but are weakly described in the current SHSPs and the FHWA’s four steps for evaluations are not often evident. Previous SHSPs have not demonstrated effectiveness during the development of current SHSPs, partly because of the difficulty associated with assessing cause and effect, and partly because they have not been subject to robust evaluations. Our assessment provides no evidence to contradict the earlier proposition that *ex post* evaluation is problematic, as there are many factors contributing to road safety 6,13,17] and the value of SHSPs has not been proven [20]. Moreover, thorough implementation is crucial to the success of SHSPs to ensure efficiency and effectiveness in achieving outcomes. FHWA guidelines describe the ‘essential eight’ components for successful implementation: four elements (1. leadership, 2. collaboration, 3. communication, and 4. data collection and analysis) and four steps (1. developing emphasis area action plans, 2. integrating the SHSP into other transportation and safety plans, 3. developing a marketing strategy, and 4. monitoring progress, evaluating results, and establishing a feedback loop to ensure SHSP adjustments and updates are continually incorporating experiences and lessons learned) [83]. Unfortunately, these eight components are not evident in most SHSPs, which consequently threatens their success. SHSPs would benefit from thorough evaluations and robust implementation plans with a commitment to complete delivery to ensure the SHSPs are properly applied. However, the opportunity to apply *ex ante* assessments would be more timely and therefore beneficial, than *ex post* evaluations that are subject to lengthy delays.

As for other similar studies [19,79] the analysis presented in this study is limited by the information available and within the scope of the review, i.e., the published SHSPs. Additional SHSP complementary information, such as actions plans, and other supporting information may also be available, but not referred to in the plans, such as in the Highway Safety Improvement Plans, Commercial Vehicle Safety Plans, or broader transport plans. For a comparative assessment, it is important that strategies are assessed on an equal basis, so including additional information, either selectively or unsystematically, can threaten the equivalence of such assessments. The style and purpose are other issues affecting the content of the plans. SHSPs may be written shorter or more simply to to improve readability for the general public. The plans may have alternative purposes, such as to engage and motivate the public, to assist decision making by elected members and agencies, or to clearly guide and direct professionals, practitioners and other participants involved. Therefore, the SHSPs may necessarily need to be written in different ways and include different content.

As this study provides a new methodology for assessing SHSPs, it is subject to further consideration and development. For instance, other assessors may prefer different criteria or different scoring for the criteria used in this study. This study relied on a single assessor to maximize consistency, but not all assessors would necessarily agree with this assessor’s score, even though the relativity of scores should remain consistent even if the nominal values change.

### 7P System criteria application

The assessment of systems criteria presented in this study (Tables 4 and 5) found that current SHSPs were strongest on one element of best practice from a system perspective (purpose), minimally adequate for some (policy tools, parts, participants, and partnerships) but weak on principles and processes. All SHSPs mentioned education, engineering, enforcement and emergency services, consistent with Federal requirements, although activities to improve emergency services were less common. Some other policy tools were occasionally included, such as incentives, taxation, leadership, integrating techniques, alternative funding and investment (e.g. private sector), subsidies, consumer choice, industry change or innovation. All SHSPs mentioned several types of road users, vehicles (mostly several types) and roads (often with the wider infrastructure). Other parts of the road safety system that affect outcomes were rarely mentioned, such as land use, the economy, social context, and safety management [6]. Emphasis or Focus Areas clearly identified specific aspects for the SHSPs to target and were a mix of parts (e.g. roads or road users), behaviors (e.g. distraction, alcohol) or activities (e.g. data, enforcement and education).

Principles to guide road safety management and practice were adequately described in 17 SHSPs, most often based on AASHTO or TZD, or were generally stated as ways of approaching the problem. However, most SHSPs did not include such clear guidance. Other valuable principles to ensure the SHSPs were cost-effective, acceptable and timely were only occasionally described, such as innovation, administrative efficiency and effectiveness, resilience to future change, national consistency, practicability or operational and commercial efficiency and effectiveness.

Processes that need to be applied in order for the intended road safety outcomes to be achieved were adequately described in 32 SHSPs although there was considerable room for improvement in almost all SHSPs. Consistent with Federal requirements, all SHSPs described the process to develop the plan, implementation and evaluation, although often weakly. Marketing, collaboration or communication was also regularly included, however, other valuable processes for safety management, research, project design and, communications, etc. were rarely or never mentioned. Additional processes to apply best practice safety management that exist in other safety domains were also missing, including in-depth crash investigation, thorough safety and risk management, scenario assessments, benefit-cost assessment, program evaluation, etc. Other contemporary risk analysis and management that could be applied includes fault tree analysis [31], Management Oversight and Risk Tree (MORT) [25], real options analysis [76], Systems Theoretic Accident Modeling and Processes (STAMP) [31,77], SAFETY II [11] and systems dynamics [31,77,78].

The development of all SHSPs involved many stakeholders, often with diverse roles and varied perspectives involving extensive collaborative participation [11,31,77]. However, these stakeholders were generally not carried over into subsequent stages of design and implementation, and other relevant participants were often not included. Implementing the multifaceted programs in the SHSPs to address complex emphasis areas would benefit from the involvement of all participants that can make a positive contribution, at the same time as minimizing interference from participants with conflicting objectives. In the same way, many SHSPs included several complementary activities together in individual emphasis areas, although elsewhere the interrelationships between different policy tools, participants and processes were often not well recognized. Analyzing, describing and understanding these partnerships provides an opportunity to maximize complementary synergies and collaborative efficiency during SHSP implementation.

All SHSPs included education and enforcement, with the primary objective being to change roaduser behavior. Traditional road safety measures rely on education and enforcement as the policy tools to change behavior by increasing compliance with rules, but these alone do not work [84]; they can be useful but are not sufficient: “*education alone does not necessarily motivate the safe choice*. *By contrast*, *transformative education addresses the cultural attitudes*, *values and beliefs surrounding a set of behaviors*, *such as driving-related practices*, *and tries to motivate change by changing the culture itself*.” ([84], p16). More sophisticated behavior change programs have been found to be more successful in health promotion and achieving other objectives in transport [85,86], including the Theory of Planned Behavior (TPB) [87,88] and the Transtheoretical or Stages of Change [89]. These more advanced programs provide an opportunity to improve road safety education and other activities that are intended to change road users’ and other participants’ behaviors.

Twenty-three SHSPs discussed the concept of TZD, while another 14 mentioned it briefly or had numerical 'Toward Zero' goals. While TZD might be a useful label for public engagement, the term has various and different meanings, rather than the concept, principles and practices described in the national dialogue described by TZD, FHWA and others. Often called Vision Zero, TZD has been developed and adopted in several European and Australasian jurisdictions but varies considerably in its description and application [17,27]. Most commonly, TZD includes clear and challenging targets, and the principles that any loss of human life is unacceptable and avoidable, that humans do make mistakes, the system should take account that humans can only absorb limited forces during crashes, and there is a shared responsibility for road safety [3,18,20]. Consequently, TZD is fundamentally a philosophy to influence policy makers, the public and designers. Therefore, TZD can potentially be effective if it is translated into strategies, plans and actions that are consistent with its principles. TZD in the U.S. positively differs from other international jurisdictions by including Safety Culture and systems approaches [83]. In the U.S. and internationally, the translation of TZD into plans continues to rely on road engineering together with driver education and enforcement, which continues the common practice of many years. While some SHSPs included TZD concepts, it was not clearly translated into actions in the SHSPs, so traditional approaches to road safety continued to dominate.

As noted above, Safety Culture is a concept that has been recognized as being valuable in safety-critical industries and has successfully been applied over a long period to change participants' behavior to achieve the intended purposes [46–51]. The application of Safety Culture to road safety has been emerging for some time [6,29,80–82] but has not become intrinsic to U.S. road safety management. Safety Culture is described as "*the shared values*, *actions and behaviors that demonstrate a commitment to safety over competing goals and demands*" [90]. Both organizational and public Safety Culture are important to achieve road safety outcomes [90]. Organizational Safety Culture can be achieved through leadership, systems, standards and processes and applies to employers, as well as departments responsible for safety management. Public Safety Culture is more difficult to achieve in the absence of authority to do so. Fifteen SHSPs discuss the concept of Safety Culture, while another 14 SHSPs mention it briefly, so it has not yet been widely applied methodically. Therefore, the application of Safety Culture for road users, professionals, other participants, decision-makers and the general public offers another potential tool to improve road safety.

While systems approaches have been widely and successfully applied in different domains, it might be foolish to assume they are complete or adequate. At the same time, there appears to be little literature that challenges systems approaches; other ways of thinking or practice or details tend to be complementary not contradictory. Once a system is described and understood, it must be recognized that it can change over time, participants and parts can be added or removed, increased or decreased and new policy tools may be developed and applied. The road system is never completely in equilibrium but is constantly changing over time. So, the dynamic nature of the road safety system must be appreciated, and its management framework must be dynamic to remain relevant. This issue is part of the reason that future situation assessment is required. While a systems approach is justifiable theoretically, it can also be difficult to apply in practice, due to its complexity. The number of participants, policy tools, parts and interactions are too numerous to take full account of [6,77], so road safety requires careful management.

### Future changes criteria application

Results of this study regarding future changes affecting road safety found that existing SHSPs hardly reflected the anticipated future context, while the changing and variable nature of future conditions was missing almost entirely from consideration and responses. Less than 10% of the SHSPs achieved an acceptable score for Future Changes criteria, with the overwhelming majority of SHSPs did not relate to future circumstances and instead reflected a historical perspective based on past circumstances and practice. Although the transport market, business models and user preferences have been evolving for many years, such changes were barely recognized. SHSPs did not assess or forecast the impact resulting from the implementation of the SHSPs, and there were no processes or techniques described to do so. The most common view of the future was to outline the path to future targets without a justification that the SHSP could achieve that outcome. Even though the future has been becoming continually more changeable, complicated and unpredictable, the SHSPs generally assumed the opposite.

While new technologies were mentioned, the comments were mainly focused on the negative impact of technology on distraction and opportunities for increased automated enforcement. There was little mention about new technologies to improve road safety directly (such as in-vehicle safety systems and driverless technology), and no clear actions to apply such technologies, particularly the introduction of driver assistance systems, to the point that driverless cars were not foreseen. Responses to the introduction of such useful technologies were almost completely absent in SHSPs, despite such technologies being deployed, and sometimes mandated today. Car automation is a major focus of government, transport and general industry and similar technologies are emerging to improve safety outcomes for pedestrians, cycling, heavy vehicle and motorcycles, which also need to be accounted for.

The low scores for Futures Criteria raise the question as to whether the criteria may be too stringent. Since SHSPs have not been subject to assessment regarding future relevance, this issue would benefit from further consideration and development of reasonable measures of acceptable criteria and scores that are broadly agreed.

### Recommendations

Based on systems theory and best practice in safety management, the following recommendations are made to improve U.S. SHSPs. Firstly, consistent with systems theory and practice [6], adoption of a comprehensive systems-based framework would include:

- clearly describing and applying principles that need to guide SHSPs to be most effective, such as those underpinning the TZD approach, cost efficiency, innovation, best practice and evaluation;
- maximizing the contributions of all relevant participants by stimulating participants who can positively contribute to road safety outcomes and minimize the effects of participants who can negatively interfere;
- adopting additional alternative policy tools that broaden the range of actions that can be applied. These may include economic incentives, developing Safety Culture and climate, or capability development and standards for participants with poorer skills and knowledge;
- identifying other component parts that can be influenced to improve road safety or counteract if they would result in adverse road safety outcomes. These could include aspects of the transport and land-use system, society or economic context including broader government policy;
- thoroughly describing and applying the most useful processes required to manage road safety properly, including risk analysis, thorough implementation and evaluation;
- identifying and maximizing the positive relationships between participants, policy tools, components and outcomes and minimize the negative influences; and
- describing the outcomes or purposes of individual actions in addition to the SHSPs as a whole, and for specific sectors (such as heavy vehicles, geographic areas, road user groups of participants).
- The following recommendations are made to ensure that the SHSPs are more suitable for future circumstances:
- identifying and taking into account influences and factors that will change in the future and affect road safety outcomes;
- developing and applying techniques to manage future influences that are uncertain to ensure that the SHSPs are resilient to alternative future situations caused by changing circumstances;
- employing contemporary futures analytical techniques, such as scenario analysis, real options analysis, and Monte Carlo simulation for analysis of future consequences caused by the SHSPs, individual actions, and external factors and participants;
- developing actions to maximize the benefits of positive influences and minimize the effects of negative influences of automation and technology, new business models and the effects of changing consumer preferences; and
- ensuring an appropriate time that SHSPs should be applied to, so they remain relevant throughout their lifespan.

Finally, the methodology used in this study could be further reviewed and refined in subsequent research.

## Summary and conclusions

This study developed a new methodology to assess SHSPs from the perspectives of comprehensive system-based safety management and relevant future issues that can be applied before an SHSP is implemented. This study demonstrates that U.S. SHSPs could be developed to more thoroughly respond to the increasingly difficult challenge to improve road safety and respond to changes in the transport, economic, social and business contexts that are occurring. The SHSPs are most strongly influenced by good processes and practices described by Federal requirements and national collaborative approaches. There is a risk that current SHSPs are developed primarily to comply with Federal requirements, rather than achieving outcomes based on high-quality safety management practices. The SHSPs and national guidance are unfortunately overly reliant on historical perspectives and do not take sufficient account of future situations, including increasing complexity, changeability and unpredictability. They could be improved by applying analytical techniques to assess future situations. Generally, SHSPs are only minimally acceptable at present and could be further improved by applying thorough approaches based on systems theory and best practice safety management that have been successful in hazardous industries. Broader structural elements of a comprehensive safety framework need to be considered more deeply and astutely incorporated. Indeed, the SHSPs should be improved so that they are more likely to be successful and better applicable to the future than current versions. Finally, the concepts and methodology in this study can be applied proactively during the development of SHSPs without waiting for lengthy periods after implementation to examine the appropriateness of plans by which time the evaluation may be out of date. Applying the learnings from this study is expected to more efficiently and effectively continue the improvements to road safety.
